# The Sanitary Condition of New York City Stables

**Published:** 1881-01

**Authors:** Allen S. Heath


### ART. V.—THE SANITARY CONDITION OF NEW YORK CITY STABLES.

In the City of New York, the animals of all kinds, for labor and for food, foot up to the enormous total, in round numbers, of six millions and a quarter (6,250,000). For the year 1879, the New York Produce Exchange furnishes the following tabulated figures: Beeves, 567,837; cows, 7,336; calves, 156,227; sheep, 1,507,739; hogs, 1,725,537. All these animals require temporary shelter and food till slaughtered. While living, the manure and filth accumulated by them will not fall short of 50,000 tons; and, including the offal after being slaughtered, the accumulations must reach the total of not far from 75,000 tons of the fittest soil for the propagation of contagious diseases. This manure and offal is widely distributed throughout the city, with a population of its own of a million of human beings, to which is added at least a third of a million of daily visitors. At the same time, and added to these food animals, New York houses and feeds 50,000 horses. The accumulations of manure and filth from these horses, in a year, amounts to 100,000 tons. For these 50,000 horses there are only 10,000 stables. Of these stables, not more than one in a hundred is in perfect sanitary condition. About one-quarter of the horses are kept in dark, damp stables, or places equally bad. These are the direct sources of most of the mortality of our city horses. As compared with the mortality of stables on the first and second floors, the mortality of the cellar stables is about as ten to one. But the diseases of the horses in the cellar stables rise to a higher ratio; for the horses in the first and second floor stables seldom suffer from rheumatism, neuralgia, malaria, and blindness, brought on by inflammatory affections of the eyes.

Horses suffer sadly from ammoniacal and other animal odors; and especially from the decomposition of all nitrogenous excreta. These produce ophthalmia and catarrhs, which deprive the owners of the use of their horses to a considerable extent, and which is a monetary loss, without taking into account the lessened working life of these horses, which is of greater importance than the temporary loss of use.

A horse or cow consumes at least seven times as much air as a man, yet only one stable in one hundred is constructed with an intelligent understanding of this indispensable demand for the health of the horse or cow.

Of the two thousand cow-stables, there are not ten which are in a sanitary respect perfect.

Of the ten thousand horse-stables, about three thousand have sewer connections. The stables of the road-horses are much better than those of the truck, cart, and other work-horses. These latter occupy mostly damp, dark, illy-ventilated stalls, the plank floors saturated with accumulations of urine and filth, which cause disease of the feet of the horse. These diseases of the feet of work horses form the most frequent cause of lameness. The poor man's horse suffers most from bad stabling, and he is the least able to bear the loss of the use of his animal.

Many of the city-kept cows sooner or later suffer from tuberculosis, because of the bad sanitary condition of the stables. The average life of the city cow is less than a third of that of the country cow.

The labor life of the city horse is about half of what it should be.

ALLEN S. HEATH, M. D.

[*To be continued.*]

